# Climate- and gateway-driven cooling of Late Eocene to earliest Oligocene sea surface temperatures in the North Sea Basin

**DOI:** 10.1038/s41598-019-41013-7

**Published:** 2019-03-14

**Authors:** Kasia K. Śliwińska, Erik Thomsen, Stefan Schouten, Petra L. Schoon, Claus Heilmann-Clausen

**Affiliations:** 1 GEUS Geological Survey of Denmark and Greenland, Department of Stratigraphy, Øster Voldgade 10, 1350 Copenhagen K, Denmark; 2 Aarhus University, Department of Geoscience, Høegh-Guldbergs Gade 2, 8000 Århus C, Denmark; 3 NIOZ Royal Netherlands Institute for Sea Research, Department of Marine Microbiology and Biogeochemistry, Utrecht University, Texel, The Netherlands; 4 Department of Earth Sciences, Faculty of Geosciences, Utrecht University, Utrecht, The Netherlands

## Abstract

During the late Eocene, the Earth’s climate experienced several transient temperature fluctuations including the Vonhof cooling event (C16n.1n; ~35.8 Ma) hitherto known mainly from the southern oceans. Here we reconstruct sea-surface temperatures (SST) and provide δ^18^O and δ^13^C foraminiferal records for the late Eocene and earliest Oligocene in the North Sea Basin. Our data reveal two main perturbations: (1), an abrupt brief cooling of ~4.5 °C dated to ~35.8 Ma and synchronous with the Vonhof cooling, which thus may be a global event, and (2) a gradual nearly 10 °C temperature fall starting at 36.1 Ma and culminating near the Eocene-Oligocene transition at ~33.9 Ma. The late Priabonian temperature trend in the North Sea shows some resemblance IODP Site U1404 from the North Atlantic, offshore Newfoundland; and is in contrast to the more abrupt change observed in the deep-sea δ^18^O records from the southern oceans. The cooling in the North Sea is large compared to the pattern seen in the North Atlantic record. This difference may be influenced by a late Eocene closure of the warm gateways connecting the North Sea with the Atlantic and Tethys oceans.

## Introduction

Following the Early Eocene Climatic Optimum, the Earth’s climate entered a phase of decreasing temperatures culminating at the Eocene–Oligocene transition (EOT) with the formation of large ice sheets on Antarctica (the Earliest Oligocene Glacial Maximum; EOGM)^1,2^. The onset of the EOGM is marked by a positive δ^18^O excursion in deep-sea benthic foraminifera close to the Chron C13r–C13n boundary and known as the Oi-1 event^2–6^. However, the δ^18^O values are influenced by both temperatures and volume of continental ice and the exact magnitude of the temperature decrease across the EOT is far from clear. Estimates based on various proxies indicate that the deep sea generally cooled between 3 ºC and 5 ºC^1,7,8^, while the surface waters cooled from less than 2 ºC to 6 ºC with large geographical variations^7–10^. Records of δ^18^O of benthic foraminifera from the southern oceans indicate that the long-term middle and late Eocene cooling was superimposed by several smaller transient temperature fluctuations. One of the most distinct of these is a brief cooling dated to ~35.8 Ma and referred to as the Vonhof cooling event^11,12^. The Vonhof cooling event has hitherto been observed mainly in southern oceans. Extra-terrestrial spherules present at the onset of the event at a number of these sites initially led to suggestions that the Chesapeake Bay and Popigai impacts triggered the cooling^11^. However, this assumption was later challenged by others (e.g. ref.^13^; see below). Until now the geological records of the Vonhof cooling event are scarce and the nature of the event is not fully understood.

Several recent temperature records that document the climatic changes in the Northern Hemisphere during the late Eocene to early Oligocene are at odds with the rather abrupt changes indicated by the deep-sea δ^18^O records from the southern oceans. A study from offshore Newfoundland shows no change across EOT^10^, while a study from the Greenland-Scotland Ridge indicates a gradual change stretching over nearly 3 Ma^9^. A recent high-resolution δ^18^O and δ^13^C benthic foraminifera study from the Atlantic Ocean, including sites from the southern Labrador Sea, argues for deep water formation sourcing from the Norwegian-Greenland Sea, which pre-dated the Antarctic glaciation^14^. Several terrestrial studies, mainly from Europe, have been carried out, but they are often ambiguous as regards the magnitude and abruptness of the temperature fall during the EOT^15–22^.

Here we reconstruct changes in surface water temperature (SST) in the eastern North Sea Basin during the late Priabonian to earliest Rupelian (38.6–33.5 Ma), utilizing the TetraEther indeX of 86 carbon atoms, TEX_86_^23–26^ (Methods). We evaluate the TEX_86_-derived temperatures in relation to δ^18^O records measured on benthic and planktic foraminifera and compare the data from the North Sea with previously published results from the Atlantic Ocean.

**Figure 1:**
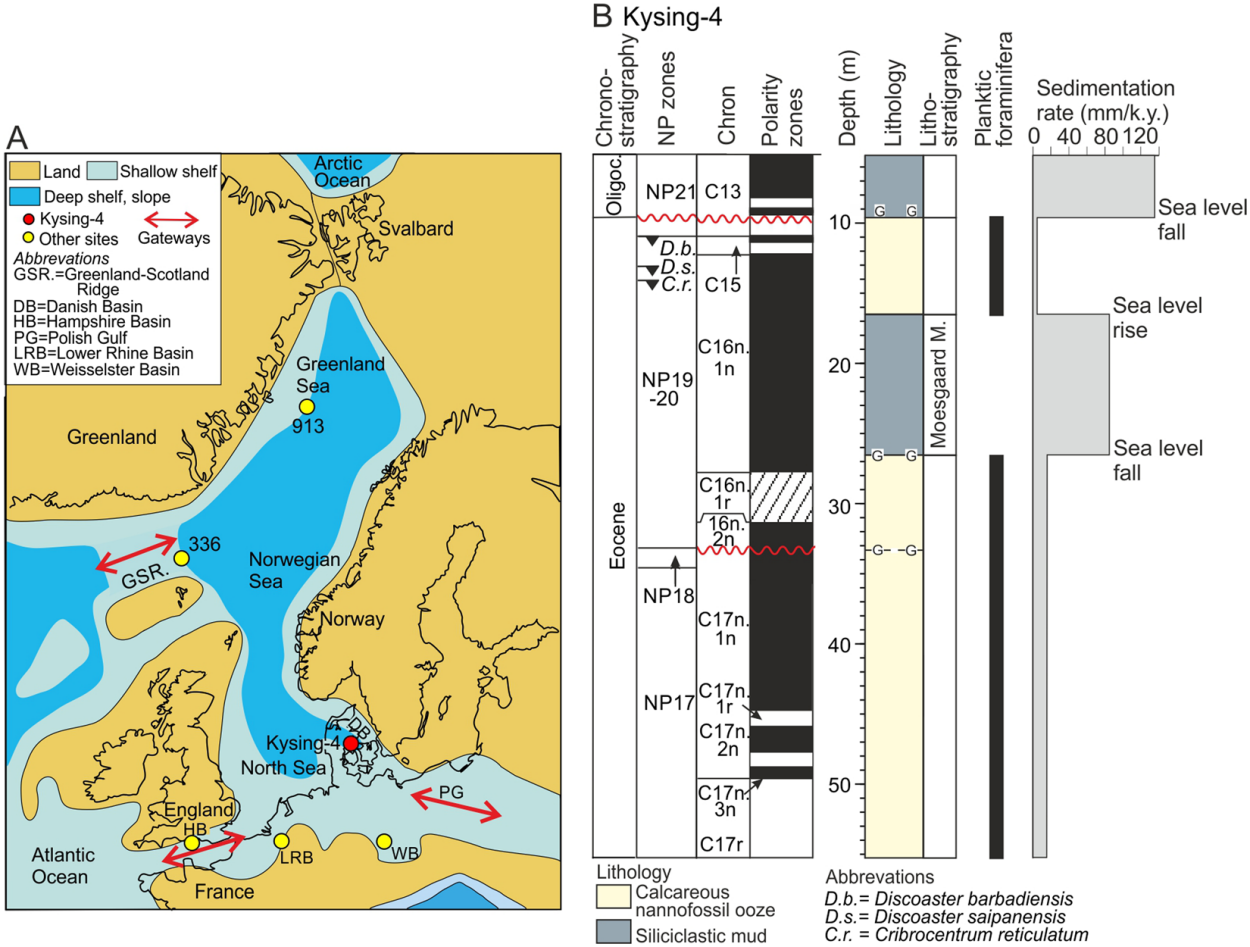
The Kysing-4 borehole and its paleogeographical position in the late Eocene semi-enclosed North Sea, Norwegian Sea, Greenland Sea system. (**A**) Location of Kysing-4 (red dot) and other sites discussed (yellow dots) shown on a late Eocene map (~35 Ma). Gateways between the North Sea, Norwegian Sea, and Greenland Sea and the world oceans are indicated. Map based on ref.^62^ www.schweizerbart.de/senckenberg and ref.^95^ (**B**) Lithology, distribution of planktic foraminifera and sedimentation rate of Kysing-4. Chronostratigraphy, calcareous nannoplankton zonation (NP zones) and magnetic polarity zones are indicated (based on data from ref.^27^).

The study is based on the well-calibrated middle Eocene (Barthonian) to earliest Oligocene (earliest Rupelian) succession in the Kysing-4 borehole located in the eastern part of the North Sea Basin^27^ (Fig. 1A). The site is unique, because it penetrates the most complete marine record of the upper Eocene to lowermost Oligocene in this part of the North Sea Basin^27^. The paleolatitude of the core site during the late Priabonian was ~50 °N cf. ref.^27^. Kysing-4 is at the moment the most northerly located site where both SST as well as benthic foraminiferal δ^13^C and δ^18^O records are available(cf. ref.^14^). During the late Eocene, the North Sea and the Norwegian-Greenland Sea formed an elongate sea connected to the world oceans through a number of shallow gateways (Fig. 1A).

The middle and upper Eocene deposits in Kysing-4 consist of fine-grained calcareous nannofossil ooze intersected by a sharply delimited 10 m thick unit of dark, coarser grained siliciclastic mud dated to Chron C16n.1n (Moesgaard Member)^27^ (Fig. 1B). The nannofossil ooze is overlain by a lower Rupelian unit of dark mud similar to the Moesgaard Member. The ooze is rich in planktic and benthic foraminifera and the lower part below the Moesgaard Member contains palynofacies strongly dominated by marine dinoflagellates^28^ and was probably deposited at a water depth of 300–400 m^27,29^. The Moesgaard Member and the Oligocene muds lack planktic foraminifera and yield a minor amount of reworked dinoflagellate cysts (up to ~3% of the total assemblage)^27,28^. The palynofacies consists of mixed marine and terrestrial particles^28^ and both units are associated with sea-level falls^27,28,30^.

## Results

**Figure 2:**
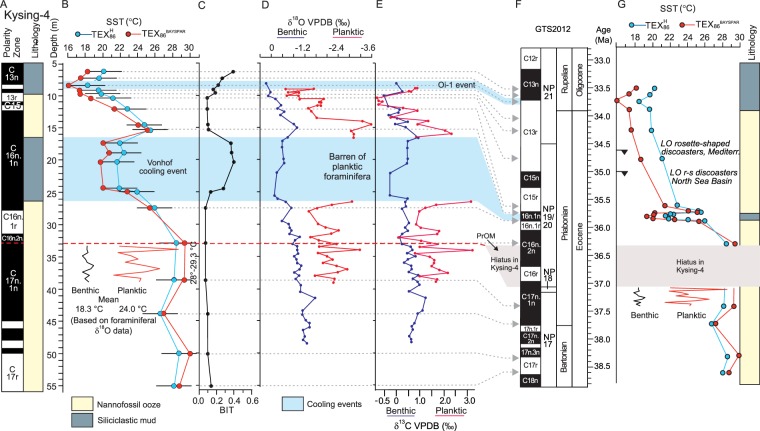
Paleotemperatures calculated for the late Eocene and earliest Oligocene interval in the Kysing-4 borehole. (**A**) Main lithology and magnetic polarity zones of Kysing-4. (**B**) Late Eocene SST record based on the TEX_86_ proxy (${\text{TEX}}_{86}^{{\rm{H}}}$^24^ and BAYSPAR^25,26^ calibrations). Error bars for ${\text{TEX}}_{86}^{{\rm{H}}}$ reflect the residual standard error^24^). Inserts in (*A*) shows thermocline and bottom water temperatures calculated from planktic and benthic δ^18^O values for the time interval 37.4–37.1 Ma, assuming 10% of present ice volume (see text). (**C**) BIT index (see text). (**D**) Planktic and benthic δ^18^O records from Kysing-4 measured on the planktic thermocline dweller *Subbotina* sp. and the benthic species *Cibicidoides eocaenicus*, respectively. (**E**) Planktic and benthic δ ^13^C records measured on *Subbotina* sp. and *Cibicidoides eocaenicus*, respectively. (**F**) Geologic time scale including polarity chrons and calcareous nannofossil zones (NP zones) from ref.^27^, updated to the geological time scale GTS2012^67^. (*G*) SST calculated from the TEX_86_ proxy plotted on the GTS2012^67^.

The Eocene calcareous nannofossil ooze is characterized by low BIT values ranging from 0.08–0.15 (Fig. 2C, Table S1) suggesting relatively low terrestrial input^31^. Our record shows two BIT excursions with maximum values of 0.4 (Fig. 2C), which both correlate with the dark, muddy units, implying a significant rise in the riverine input from land (Fig. 2C).

Regardless of the TEX_86_ calibration, the sea surface temperature (SST) records show overall similar patterns (Fig. 2B,G). The TEX_86_–derived SST shows stable high values of ~28 °C (using the calibration in ref.^24^) during the late Bartonian and early Priabonian (38.6–36.1 Ma) and low values of ~15–22 °C during the late Priabonian and early Rupelian (35.6–33.75 Ma) (Fig. 2B,G; Table S1). The temperature decline from middle Priabonian (~36.1 Ma) to earliest Rupelian (~33.7 Ma) is over 10 °C (from ~28 °C to ~15 °C). The most striking feature in our record is a distinct transient temperature minimum (of ~mean SST_TEX86H_ _=_ 20.5 °C or SST_BAYSPAR_ = 22.5 °C) corresponding roughly with the Moesgaard Member. This dark siliceous mud unit was deposited during the early part of Chron C16n.1n over a period of approximately 100,000 years^27^. The C16n.1n cooling is followed by a brief recovery (to ~25 °C) in the late part of Chron 16n. From here the temperatures gradually decrease to a minimum (~15 °C or 19 °C) during the earliest Oligocene (latest Chron C13r).

The trend of the benthic foraminiferal δ^18^O record closely follows the fluctuations shown by the TEX_86_-derived SST (Fig. 2D). The planktic δ^18^O record also shares basic similarities with the record based on organic proxies, despite a gap across the Moesgaard Member, which is barren of planktic foraminifera (Fig. 2D,E). The δ^13^C record is virtually a mirror image of the δ^18^O record (Fig. 2E). The noisy appearance of the δ^13^C and δ^18^O data between 37.3 and 35.7 Ma is attributed to Milankovitch cycles. Milankovitch cycles are present in most of the core^27^, but they are not a part of this investigation.

## Discussion

### North Sea temperatures during the early Priabonian

The TEX_86_ SST and the benthic foraminiferal oxygen isotope records are very similar and suggest that the δ^18^O values were primarily controlled by the temperature (Fig. 2). However, the oxygen isotope composition of the ambient water has also a significant influence on the δ^18^O values of foraminiferal carbonate, and several studies indicate that the δ^18^O_water_ of the world oceans fluctuated during the late Eocene due to the build-up of transient continental ice-sheets on Antarctica^4,32^. These global changes also affected the North Sea as indicated by the paleontological and sedimentological shifts that characterize the upper Eocene and lower Oligocene deposits of Kysing-4. In this study we refrain from using the δ^18^O values as a temperature proxy except for the lowermost sequence deposited between 37.4 and 37.1 Ma (late Chron C17n.1n) (Fig. 2B,G). This part of the succession was probably deposited in a stable open marine environment as indicated by the uniform sedimentary facies and micropaleontology^27,28^. We calculated water temperatures for two ice-sheet scenarios; one with 10% of current ice-sheets present at the poles and one with 40%. In the calculations, it is assumed that the sea level in an ice-free world would be 66 m higher than today^33^. For the time interval 37.4–37.1 Ma, TEX_86_-derived temperatures are ~28 °C, while temperatures based on planktic and benthic δ^18^O values are somewhat lower (24.0 °C and 18.3 °C, respectively, for a world with 10% of the present continental ice volume (Fig. 2B,G), and 25.1 °C and 19.3 °C, respectively, for a world with 40% of the present ice volume), but in view of the proxy errors (ca. 2.5 °C for TEX_86_ and ca. 1 °C for oxygen isotopes) they are quite close. The planktic oxygen isotope values are measured on *Subbotina* sp., which is considered to be a thermocline-dweller^34,35^ and thus probably records a subsurface temperature. The higher temperatures indicated by the TEX_86_ proxy in the Kysing-4 record thus seem to reflect the temperatures of the upper mixed layer in this particular case, although it has often been shown to reflect subsurface temperatures (ref.^36^ and references cited therein). Indeed, the difference between the TEX_86_ and the δ^18^O based temperatures of 3–4 °C is within the range of the modern observations of the difference between surface and thermocline temperatures^34,35^. The temperatures estimated for the bottom water are ~6 °C lower than temperatures for the thermocline (Fig. 2). This difference seems plausible with a probable water depth of 300–400 m^27,29^.

**Figure 3:**
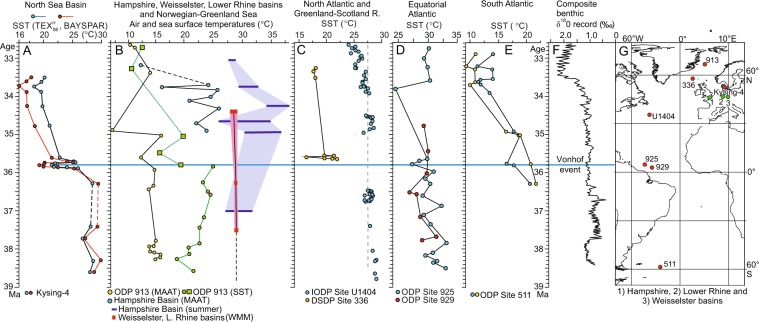
Comparison of North Sea Basin temperatures with selected, previously published temperature records. All ages are updated to GTS2012^67^. (**A**) Kysing-4 section (this study). (**B**) Hampshire Basin: mean annual air temperatures (blue dots)^22^, and summer temperatures of freshwater (blue bars)^18^. Weisselster and Lower Rhine basins: warm month mean temperatures (red bars)^17,20^. Norwegian–Greenland Sea: mean annual air temperatures (soil bacteria lipids, yellow dots)^15^; sea surface temperatures ${\text{TEX}}_{86}^{{\rm{H}}}$ derived (green dots)^38^ and alkenone-derived (green squares)^9^. (**C**) North Atlantic sea surface temperatures (alkenones, blue dots)^10^. Greenland–Scotland Ridge sea surface temperatures (alkenones, yellow dots)^9^. (**D**) Equatorial Atlantic sea surface temperatures (${\text{TEX}}_{86}^{{\rm{H}}}$)^38^. (**E**) South Atlantic sea surface temperatures (TEX_86_, blue dots; alkenones, yellow dots)^9^. (**F**) Composite benthic δ^18^O record^67^. (**G**) Locality map. Modern coastline shown on a 35 Ma plate tectonic reconstruction. Map created using the OSDN *Plate Tectonic Reconstruction* Service.

The TEX_86_-derived temperature of ~28 °C for the early Priabonian (until ~36.2 Ma) in Kysing-4 agrees well with contemporaneous, terrestrial summer temperatures of 27–32 °C estimated for the Hampshire Basin, southern England^18,22^ (Fig. 3B) and with terrestrial summer temperatures of 27–28 °C estimated for the Lower Rhine and Weisselster basins^17,20,37^ (Fig. 3B). It is also in agreement with the early Priabonian sea surface temperatures of ~26–28 °C derived from alkenones from the northwest North Atlantic^10^ (Fig. 3C), with sea surface temperatures of 23–24 °C calculated for the central Greenland Sea using the TEX_86_ proxy^38^ and with mean annual air temperatures of about 13–15 °C estimated for the adjacent land area in East Greenland on the basis of soil bacteria lipids^15^ (Fig. 3B) and spore-pollen assemblages^16^. The temperature difference between Kysing-4 and central Greenland Sea is smaller than today, but it is in accord with the lower Eocene latitudinal temperature gradient e.g. ref.^39^.

The overall agreement between the TEX_86_ -derived SST and the terrestrial records may suggest a seasonal bias of the TEX_86_ proxy in our record towards summer. A similar summer bias has been observed in other TEX_86_ studies, although the proxy mostly is considered to represent a mean annual temperature (see discussion in ref.^36^). Foraminiferal δ^18^O records seem generally also to reflect summer temperatures^40^.

### The EPi-1/PrOM cooling event

In all investigated upper Eocene sections in Denmark, including Kysing-4, NP18 is missing or very thin indicating the presence of a hiatus^41,42^. In Kysing-4, the hiatus comprises the upper part of C17n.1n to the lower part of C16n.2n (Fig. 2). The Danish hiatus can be correlated with the Belgian Bassevelde 1 depositional sequence, which is of very limited extent as compared to sequences below and above^43,44^. The Bassevelde 1 sequence is correlated with the European–North American Bart2/Pr1 sequence of Hardenbol *et al*.^43,44^. The sea-level fall associated with the major sequence boundary at the base of the Bart2/Pr1 sequence is associated with a positive δ^18^O excursion recorded in Chron C17n.1n at ODP Site 689 (Southern Ocean) and named the EPi-1 event^45,46^. More recently, a cooling event based on a benthic foraminiferal δ^18^O excursion tentatively placed in Chron C17n.1n (also based on correlation with ODP Site 689) has been indicated in the Southern Ocean (ODP Site 738)^47^. The event was named the PrOM-event and appears to be the same as the EPi-1 event.

In the benthic foraminiferal δ^18^O record of Kysing-4 there is no indication of a positive excursion in the preserved part of C17n.1n (Fig. 2) and it is most probable that the EPi-1/PrOM event falls within the regional hiatus comprising the upper part of C17n.1n and the lower part of C16n.2n, thus supporting previous suggestions connecting the event to a glacioeustatic sea-level fall.

### The Vonhof/C16n.1n cooling event

The most distinct climatic event in the 4 million years long record is a transient (100,000 years) cooling dated to ~35.8 Ma (Chron 16n.1n) (Fig. 2G). The onset of the cooling is marked by a SST drop of ~5–8 °C (Fig. 2). The cooling coincides with a shift from hemipelagic nannofossil ooze to siliciclastic mud (Moesgaard Member), with elevated BIT values (Fig. 2C), and with an increase in the amount of terrestrial organic particles^28^, altogether indicating a significant sea-level fall^27–29^ (Fig. 1). The cooling is also observed in the benthic δ^18^O record, which shows an increase of about 0.7‰, closely following the trend of the TEX_86_–derived SST (Fig. 2).

The combination of a brief sea-level fall, a drop in δ^18^O, and a significant decrease in SST is most simply explained by an increase in the volume of continental ice, causing a glacio-eustatic, hence global, sea level fall. Using preliminary δ^18^O data a glacio-eustatic model has previously been proposed for the deposition of the Moesgaard Member^27,30^. In both of these studies, the Moesgaard Member was correlated with the Vonhof cooling event^11,12^. The Vonhof event is marked by a distinct increase in the δ^18^O values of benthic foraminifera in several ODP boreholes from the high latitude southern oceans^11,12^. The event is less prominent in the benthic δ^18^O compilation of ref.^48^ (Fig. 3F). We observe that sea surface temperatures of sites 925 and 336 (Fig. 3C,D), although of low resolution, also show a decrease that may correspond to the Vonhof/C16n.1n event in the North Sea.

Because of a peak of extra-terrestrial spherules at the base of the event in several ODP boreholes^9,11,49^ and also in the Massignano section, Italy^50,51^ the Vonhof cooling event has been linked to the Chesapeake Bay and the Popigai impact events^11^. However, the potential cooling effect of these two impacts is far from clear as the spherule layer in several cores is associated with short-term temperature rises^13,52–54^. No spherules were detected in Kysing-4.

The glacio-eustactic nature of the sea-level fall and the cooling in the North Sea Basin suggests that the event is connected to formation of ephemeral Antarctic ice sheets. Based primarily on sea-level records and on the distribution of ice-rafted debris (IRD), several studies have argued that the southern, and possibly also the northern high latitudes, experienced short-lived glaciations through most of the late Eocene and possibly also the late middle Eocene^32,47,55–58^. These interpretations are supported by recent studies from the Antarctic Realm, indicating the presence of mountain glaciers on Antarctica from 35.8–34.8 Ma^57^, and calving glaciers along the coastline of the Weddell Sea at least going back to 36.5 Ma^59^. The occurrence of IRD in upper Eocene and lower Oligocene deposit from the Norwegian-Greenland Sea suggests that glaciers possibly formed also in the mountains of northeast Greenland^16,56^.

### The late Priabonian to early Oligocene temperature trend in the North Sea Basin

In Kysing-4, the sea surface started to cool at ~36.1 Ma based on the TEX_86_ record (Fig. 2B). Apart from the brief Vonhof/C16n.1n cold event, the temperature decrease was gradual reaching a minimum in the uppermost part of Chron C13r at 33.7 Ma. The temperature minimum probably corresponds to the global Oi-1 event^3,4^ (Fig. 2B,G). The total temperature fall during the ~2 Ma cooling period amounts to 10 °C using the ${\text{TEX}\,}_{86}^{{\rm{H}}}$ calibration and 13 °C using the BAYSPAR calibration. A gradual cooling can also be deduced from the palynofacies, which shows a significant increase in the proportion of conifer pollen in the uppermost 2 m of the Eocene ooze^28^, that is from about 35.6 Ma.

Most inferences of the climatic development at the Eocene–Oligocene transition are based on deep-sea oxygen isotope records from the southern oceans. These records generally exhibit a relative abrupt shift with majority of the changes occurring during the EOT from ~34 to ~33.4 Ma^60^. Independent temperature proxies are few, but a study from the Kerguelen Plateau in the Southern Ocean based on the Mg/Ca temperature proxy yielded an abrupt 2–3 °C cooling in deep surface waters at ~34 Ma^7^. However, not all existing records show an abrupt shift and several studies of sea surface temperatures show a more gradual cooling trend or no cooling at all. Below we compare the TEX_86_ temperature records from Kysing-4 with some of the most detailed surface water records from the Atlantic Ocean and Norwegian-Greenland Sea (Fig. 3).

The overall cooling trend indicated in ODP Site 511 from the South Atlantic^9^, although of low resolution, show similarity to the pattern in Kysing-4 regarding both the time span and the magnitude (Fig. 3A,E,G). At Site 511, similar to our record, the temperature decreases by ~10 °C between ~36.3 and 33.6 Ma. The temperature record at IODP Site U1404 from the eastern North Atlantic (offshore Newfoundland), shows a very different pattern from that of Site 511 (Fig. 3E). The temperature fall is minor (~2 °C), very gradual, and there is no evidence of a surface cooling directly coinciding with the EOT. Liu *et al*.^10^ considered the surface water temperature records of sites 511 and U1404 as representative of the southern and northern Atlantic Ocean, respectively. The sea surface temperature record of ODP Site 913 from the central Norwegian-Greenland Sea is not considered here, as the data covering our time frame are from two independent proxies, with no overlap interval (Fig. 3B). Kysing-4 show both similarities and differences relative to the North Atlantic Site U1404 (Fig. 3A,C). The main similarities between these two sites are the overall gradual cooling patterns during the late Priabonian and the lack of a significant temperature fall across the EOT. The main difference is in the magnitude of the total cooling ranging from only ~2 °C in U1404 to at least 10 °C in Kysing-4. The differences may be related to the semi-enclosed nature of the Norwegian-Greenland Sea – North Sea system. During the Priabonian, these interconnected basins were only connected to the outside oceans through shallow seaways (Fig. 1A), which during the Priabonian were affected by eustatic sea-level changes and plate tectonic movements (see below). A seaway corresponding approximately to the location of the English Channel today connected the Hampshire–Dieppe Basin in the southwestern North Sea Basin with the warm waters of the eastern North Atlantic (Fig. 1A). It was open during most of the Bartonian as indicated by the presence of marine sediments in the Hampshire Basin^55^. The Priabonian and lowermost Rupelian deposits are marginal marine and non-marine indicating that the seaway became more restricted at that time. King^29,61^ suggested that the connection was closed from the mid Priabonian.

A wide southeastern seaway between the North Sea and the warm Tethyan Realm was also severed during the late Priabonian (Fig. 1A) as a result of a combination of uplift of the Alpine–Carpathian foldbelt and the eustatic sea-level fall^62,63^. The connection between the Norwegian-Greenland Sea and the North Atlantic Ocean across the Greenland-Scotland Ridge was probably of minor importance during the late Priabonian as the sill depth over the Scotland-Greenland Ridge was only 30–50 m^64^. The initial separation of Greenland from the Svalbard area began at ~35 Ma^65^. However, the spreading zone presumably remained emerged until ~25 Ma, when shallow-water exchange became possible^66^.

Altogether, it appears that the connections to the warmer waters of the Atlantic Ocean and the Tethys Ocean became closed during the latest Eocene, while the shallow seaway to the Atlantic across the Greenland-Scotland Ridge remained unchanged. A connection to the Arctic Ocean at that time was apparently not yet established. We suggest that the closure of these connections may have influenced the development in the North Sea thus accentuating the temperature decrease in Kysing-4 during the late Priabonian.

The limited connection between the North Sea and the North Atlantic is supported by a new study from the southern Labrador Sea (ODP Site 647)^14^. However, a comparison between the two regions is difficult as the two records represent different paleodepths: 2000–3000 m in the Labrador Sea as compared to 300–400 m for the North Sea and planktic foraminifera at Site 647 are sparse^14^.

One of the more remarkable observations at Site 647 is the unusually low benthic δ^13^C values during the late Eocene^14^. They are on an average 0.5–1‰ lower than at all more southerly Atlantic sites. The late Eocene δ^13^C values of Kysing-4, including both the benthic and the planktic records, are significantly more positive (Fig. 2E) and are more in line with values from sites in the southern oceans. Judging from the δ^13^C records, it appears that the development of water masses and productivity in the two regions during the late Eocene were very different.

The δ^18^O records of benthic foraminifera indicate that there are also similarities between the North Sea and the Labrador Sea. The onset of the long-term cooling in the North Sea at ~36.4 Ma (Fig. 2G) coincides with the beginning of a long-term increase in the benthic δ^18^O values at Site 647. Increasing δ^18^O values are generally indicative of decreasing temperatures, but as oxygen isotopic composition is also affected by salinity, and thus the magnitude of the temperature decrease at Site 647 is uncertain. According to ref.^14^, the start of the increasing benthic δ^18^O values in the Labrador Sea coincides with the beginning of deep water formation in the northwest Atlantic and probably also with a sea surface warming. To which degree these oceanographic changes influenced the development in the North Sea remains unclarified.

## Methods

### Age Model

We apply the existing age model for the Kysing-4 borehole^27^ updated to the geological time scale GTS2012^67^. The upper Priabonian to lower Rupelian interval in the Kysing-4 borehole is relatively condensed with a rather weak magnetic signal^27^ (Fig. 2). Potential uncertainties in the age model for this critical interval are evaluated below.

In the upper Priabonian deposits of Kysing-4 we observe two important nannofossil events, namely the last occurrences (LO) of the two rosette-shaped discoasters, *Discoaster barbadiensis* and *D*. *saipanensis*^27^ (Fig. 2B,E,G). These two species are generally considered to disappear almost simultaneously^68,69^, but the extinction events have been shown to occur considerably earlier in high latitudes than in low latitudes^70^. In the Massignano section (Italy), the LO of the two species is in the lower third of Chron C13r^51,54^ at a level dated to ~34.6 Ma in the GST2012^67^. In Kysing-4, the LO of the group (here *D*. *barbadiensis*) occurs close to the Chron C15n-C13r boundary at a level with an estimated age of ~35 Ma. Considering the diachrony of the LO of the group, the observations in Kysing-4 are in good agreement with the observation from the Italian section and clearly supports the age model of ref.^27^.

### Organic proxies

20 sediment samples were collected from the interval between 6.5 m and 55.08 m. The total lipid extract was obtained from mechanically powdered and freeze-dried samples with the accelerated solvent extraction (ASE) technique using dichloromethane/methanol (9:1 [v/v]). The lipid extract was separated over an activated Al_2_O_3_ column into an apolar (hexane/dichloromethane; 9:1 [v/v]) and a polar (dichloromethane/methanol; 1:1 [v/v]) fraction. The polar fraction, prior to GDGT analysis, was dissolved in hexane–isopropanol (99:1 [v/v]) and filtered over a 0.4 µm polytetrafluoroethylene filter.

The distribution of glycerol dialkyl glycerol tetraethers (GDGT) was analysed by high performance liquid chromatography/mass spectrometry as described in ref.^71^. Briefly, an Agilent (Palo Alto, CA, USA) 1100 HPLC high-sensitivity mass-selective detector (MSD SL) was used. Compounds were separated using a Prevail cyano column (150 × 2.1 mm; 3 µm; Alltech, Deerfield, IL, USA) maintained at 30 °C. GDGTs were eluted isocratically with 99% hexane and 1% isopropanol for 5 min, followed by a linear gradient to 98% hexane and 2% isopropanol at a flow rate of 0.2 mL/min. Detection was achieved using single-ion monitoring. Relative qualification of the compounds was achieved by manual integration of the peaks in the mass chromatograms in the Agilent ChemStation manager software. In order to evaluate the source and the distribution of GDGTs, we calculated a number of indices: the BIT index^72^, % GDGT-0^73^, the Methane Index^74^, the *f*_*cren*′:*cren*′+*cren*_ index^75^ and the Ring Index vs TEX_86_^76^. The results imply that in all sediments ammonia-oxidizing Thaumarchaeota are the main source of GDGT. For sea surface temperature estimations, we applied the ${\text{TEX}}_{86}^{{\rm{H}}}$^24^ and BAYSPAR calibrations^25,26^. Out of 20 samples, nine were analysed in duplicate and two in triplicate. All the results are shown in Table S1.

### The TEX_86_ as sea surface temperature (SST) proxy

The TetraEther indeX of 86 carbon atoms (TEX_86_) is an organic paleothermometer, which is based on the distribution of the isoprenoid glycerol dialkyl glycerol tetraethers (isoGDGT)^23^. The original definition for calculating TEX_86_ is as follows:

S1 $$TE{X}_{86}=\frac{(GDGT-2+GDGT-3+GDGT-crenarchaeol)}{(GDGT-1++GDGT-2+GDGT-3+GDGT-crenarchaeol)}$$

IsoGDGTs are membrane lipids spanning the cell membranes of archaea. One of the most ubiquitous isoGDGTs is crenarchaeol characterized by presence of a cyclohexane moiety. Crenarchaeol is produced by the marine archaea belonging to the phylum Thaumarchaeota e.g.^77,78^. The Thaumarchaeota also synthesize other common isoGDGTs: GDGT-0 (with no cyclopentane moiety) and GDGT with 1 to 3 cyclopentane moieties. Structures of isoGDGTs are shown on Fig. S1a. Studies on Thaumarchaeota suggest that many of them are chemoautotrophs and ammonia oxidizers e.g.^79,80^.

Schouten *et al*. (ref.^23^) recognized that temperature is the main factor influencing the distribution of the sedimentary GDGTs. However, several studies recognized that the distribution of GDGTs can be influenced by other, non-thermal factors, such as: terrestrial input, oxic degradation or thermal alternation. Therefore, in order to ensure that GDGTs origin from ammonia-oxidizing Thaumarchaeota, it is important to evaluate the distribution and source of the GDGT for potential bias. For that purpose, we have utilized a number of indices.

### The branched and isoprenoid tetraether (BIT) index

The BIT index is calculated as a ratio between the branched GDGTs (brGDGTs are synthesized by soil and river bacteria; for more see e.g.^36^ and references cited therein) versus crenarchaeol. The index values are calculated as described in ref.^72^:

S2 $$BIT=\frac{({\rm{GDGT}}-{\rm{Ia}}+{\rm{GDGT}}-{\rm{IIa}}+{\rm{GDGT}}-{\rm{IIIa}})}{({\rm{GDGT}}-{\rm{Ia}}+{\rm{GDGT}}-{\rm{IIa}}+{\rm{GDGT}}-{\rm{IIIa}}+{\rm{crenarchaeol}})}$$

The roman numerals refer to individual brGDGT structures (for details see ref.^36^). Structures of brGDGTs are shown on Fig. S1b. The index aims to estimate the terrestrial input of the GDGT pool in marine environments and serves as a proxy for the relative input of soil and river organic material into marine settings^72,81–83^. BIT values span from close to 0 (absence of brGDGTs, typical for open marine environments) to 1 (absence of crenarchaeol, characteristic for mineral soils and peat)^36,72^. It is generally accepted, that TEX_86_ estimates where BIT > 0.3 can potentially be influenced by soil-derived GDGT signal, and thus should not be used for SST reconstruction^31^. However, this depends on the particular location, i.e. the TEX_86_ value of the terrestrial GDGTs transported to the marine environment as well as the mass spectrometer settings (see discussion in ref.^36^).

The BIT values are between 0.1 and 0.4, with a mean value of 0.2 (Table S1). We use a cut-of value of 0.4 and thus include all samples in further analysis.

### The %GDGT-0 index

The ammonia-oxidizing Thaumarchaeota may not be the only source of GDGTs in the marine settings. GDGT-0 and smaller quantities of GDGT-1, GDGT-2 and GDGT-3 can be synthesized by other archaea including sedimentary methanogenic archaea. In some settings, the methanogenic GDGTs can be substantial e.g.^84,85^ and therefore can bias the TEX_86_. For constraining a methanogenic input of GDGTs Sinninghe Damsté *et al*.^73^ suggested applying the %GDGT-0 index:

S3 $$ \% GDGT-0=(\frac{{\rm{GDGT}}-0}{{\rm{GDGT}}-0+{\rm{crenarchaeol}}})\times 100$$

Studies on enrichment cultures of Thaumarcheota suggest that when %GDGT-0 values reach values above 67% the sedimentary GDGT pool may be affected by an additional (probably methanogenic) source of GDGTs. Our %GDGT-0 values range between 28.8 and 48.3 with a mean value of 43 (Table S1) suggesting that the GDGT pool is most probably not influenced by methanogenic GDGTs.

### The Methane Index (MI)

It has also been suggested that some of the GDGTs preserved in the sediments may be produced by methanotropic Euryarchaeota (ref.^36^ and references cited therein). This is especially observed in settings where gas-hydrate-related anaerobic oxidation of methane is taking place^74^. To identify the methanotrophic source of GDGTs, Zhang *et al*.^74^ proposed the Methane Index (MI), which is calculated using the formula:

S4 $$MI=\frac{({\rm{GDGT}}-1+{\rm{GDGT}}-2+{\rm{GDGT}}-3)}{({\rm{GDGT}}-1+{\rm{GDGT}}-2+{\rm{GDGT}}-3+{\rm{crenarchaeol}}+\mathrm{crenarchaeo}{\rm{l}}^{\prime} )}$$

For SST calculations, it is recommended to exclude all samples where MI > 0.5. In our material MI varies from 0.18 to 0.25, with mean value of 0.21 (Table S1) and thus suggest no input of methanotrophic Archaea.

### The Ring Index vs TEX_86_

Prior to calculating TEX_86_-SST proxy it is also crucial to eliminate samples which may have been influenced by non-thermal factors and/or deviate from modern analogues e.g.^76^. To achieve that, Zhang *et al*.^76^ proposed the Ring Index (RI), which is calculated as follows:

S5 $$\begin{array}{c}RI=0\times (\frac{GDGT-0}{{\sum }^{}GDGT})+1\times (\frac{GDGT-1}{{\sum }^{}GDGT})+2\times (\frac{GDGT-2}{{\sum }^{}GDGT})\\ \,+3\times (\frac{GDGT-3}{{\sum }^{}GDGT})+4\times (\frac{crenarchaeol}{{\sum }^{}GDGT})+4\times (\frac{crenarchaeol^{\prime} }{{\sum }^{}GDGT})\end{array}$$

Where:

S6 $$\begin{array}{c}{\sum }^{}GDGT=GDGT-0+{\rm{GDGT}}-1+{\rm{GDGT}}-2+{\rm{GDGT}}-3\\ \,+{\rm{crenarchaeol}}+\mathrm{crenarchaeo}{\rm{l}}^{\prime} \end{array}$$

The formula for RI estimates a weighted average of the ring numbers in GDGT compounds. Zhang *et al*.^76^ demonstrated that in the modern core-top dataset, RI and TEX_86_ are significantly correlated. This strong relationship is expressed as:

S7 $$R{I}_{TEX}=-0.77(\pm 0.38)\times TE{X}_{86}+3.32(\,\pm \,0.34)\times {(TE{X}_{86})}^{2}+1.59(\,\pm \,0.10)$$

Zhang *et al*.^76^ furthermore suggest that TEX_86_-SST values deviating by more than |0.3| from the modern TEX_86_-RI relationship should be excluded, as they may by impacted by non-thermal factors^75,76^.

S8 $${\rm{\Delta }}\mathrm{RI}=R{I}_{TEX}-R{I}_{sample}$$

The ΔRI values in our dataset are between −0.17 and 0.10 with the mean value of 0.0 (Table S1) suggesting that the TEX_86_ follows modern day behaviour.

### The relative abundance of crenarchaeol isomer f_Cren′:Cren′ + Cren_

In order to identify anomalous GDGT distributions O’Brien *et al*.^75^ suggested a new ratio:

S9 $${f}_{Cren^{\prime} :Cren^{\prime} +Cren}=(\frac{\mathrm{crenarchaeo}{\rm{l}}^{\prime} }{{\rm{crenarchaeol}}+\mathrm{crenarchaeo}{\rm{l}}^{\prime} })$$

The ratio in our dataset is between 0.03 and 0.09 (Table S1) which is close to the lower values of the modern (0.00–0.16) core-top sediments^75^.

### TEX_86_ calibration

The first calibration of TEX_86_ as SST proxy was linear^23^. Following this, Kim *et al*.^24^ presented two logarithmic calibrations, ${{\rm{TEX}}}_{86}^{{\rm{H}}}$ and ${{\rm{TEX}}}_{86}^{{\rm{L}}}$, where ${{\rm{TEX}}}_{86}^{{\rm{L}}}\,$is more applicable in high latitude settings. Considering the mid latitude setting for our site we calculated ${{\rm{TEX}}}_{86}^{{\rm{H}}}$ values using the calibration given in ref.^24^:

S10 $${{\rm{TEX}}}_{86}^{{\rm{H}}}=\,\mathrm{log}(\frac{({\rm{GDGT}}\mbox{--}2+{\rm{GDGT}}\mbox{--}3+\mathrm{crenarchaeo}{\rm{l}}^{\prime} }{{\rm{GDGT}}\mbox{--}1+{\rm{GDGT}}\mbox{--}2+{\rm{GDGT}}\mbox{--}3+\mathrm{crenarchaeo}{\rm{l}}^{\prime} })$$

Raw ${{\rm{TEX}}}_{86}^{{\rm{H}}}$ values for the studied sediments are between 0.51 and 0.72 with mean value of 0.59.

Sea surface temperatures were calculated as follows:

S11 $${\rm{Temp}}[^\circ {\rm{C}}]=68.4\,({{\rm{TEX}}}_{86}^{{\rm{H}}})+38.6$$

Samples analysed in duplicate show reproducibility better than 0.5 °C and in most cases better than 0.25 °C (Table S1). The residual standard error for the ${\text{TEX}\,}_{86}^{{\rm{H}}}$ calibration model is 2.5 °C^24^.

One of the most recent approaches is based on a spatially varying, TEX_86_ Bayesian regression model (BAYSPAR)^25,26^. BAYSPAR model SST predictions were obtained from the online GUI at http://bayspar.geo.arizona.edu using the modern-day coordinates for the Kysing site (56.0107° N, 10.2566° E). For the “deep-time” calibration we have applied the mean of the tolerance which is equal to the mean of the TEX_86_ value (mean = 0.59), see Table S1. The prior standard deviation is set as default (i.e.^25^). The search tolerance is expressed as twice the standard deviation of the inputted TEX_86_ data (STDEV.P = 0.05973). The number of iterations to perform at each analogue site is set as default (i.e.=2000). Modern analogues for our dataset suggest low to mid latitudinal settings (Fig. S2).

The TEX_86_-derived SST for the studied interval range between 18.8 °C and 28.8 °C (±2.5 °C) for ${{\rm{TEX}}}_{86}^{{\rm{H}}}$ (Fig. 2), 15 °C and 36 °C (±5.8 °C to 8.2 °C) for BAYSPAR (Figs 2 and S3). Regardless of the calibration, the SST record derived both calibrations shows the same trend and reveals two minima. The ΔSST between the two calibrations (ΔSST = SSTBAYSPAR-SSTTEX_86_H) is below 2.6 °C (Table S1), with mean value of −1.5 °C.

The TEX_86_ has also been shown to be reflecting subsurface rather than SST e.g.^86,87^. However, since this setting is relatively shallow we assume that the trends mostly reflect upper water column conditions rather than deep water. Indeed, recent studies show that TEX_86_ gives reasonable SST estimates with respect to other proxies such as Mg/Ca and Δ47 of planktic foraminifera^88^.

Finally, TEX_86_ has been suggested to be affected by ammonium oxidation rates and/or oxygen depletion, i.e. increasing values with decreasing oxygen concentrations and ammonium oxidation rates^89,90^. However, since we do not find large changes in productivity and redox condition based on dinocyst assemblages, palynofacies and ichnofabric, we assume these factors did not have a large impact on our temperature trends.

### Inorganic proxies

#### Bottom water and thermocline temperatures derived from δ^18^O data

Planktic foraminiferal δ^18^O and δ^13^C composition was measured on *Subbotina* sp., while the benthic values were measured on *Cibicidoides eocaenicus*. *Subbotina* sp. constitutes mostly 80–100% of the planktic fauna and is the only planktic taxon which is continuously present. The second-most important planktic taxa, *Acarinina*, is only present in three short intervals^27^. The foraminifera were picked from the 100–500 µm size fractions in 52 samples of planktic foraminifera and 42 samples of benthic foraminifera. The generally well-preserved tests were crushed and ultrasonically washed in distilled water. The measurements were performed on a Finnigan MAT 253 mass spectrometer versus VPDB. The temperature reconstructions were calculated applying the equation of Shackleton^91^:

S12 $${\rm{T}}=16.9\mbox{--}4.38({{\rm{\delta }}}^{18}{{\rm{O}}}_{{\rm{calcite}}}\,\mbox{--}\,{{\rm{\delta }}}^{18}{{\rm{O}}}_{{\rm{water}}})+0.10{({{\rm{\delta }}}^{18}{{\rm{O}}}_{{\rm{calcite}}}\mbox{--}{{\rm{\delta }}}^{18}{{\rm{O}}}_{{\rm{water}}})}^{2}$$

We estimated a δ^18^O_water_ value of ca. 0.3‰ based on the modern value for the study area^92^ and corrected for changes in continental ice volume. We applied a correction factor of 0.011‰ per meter sea-level change^93^. Finally, we added 0.27‰ for conversion from the VSMOW scale to the VPDB scale^94^.

## Supplementary information

Supplementary figures
