# Supplementary material for: Climate- and gateway-driven cooling of Late Eocene to earliest Oligocene sea surface temperatures in the North Sea Basin
Source: Sci Rep. 2019 Mar 14;9:4458. doi: 10.1038/s41598-019-41013-7 (PMC6418185; doi:10.1038/s41598-019-41013-7)
Supplement: Supplementary file 1 — Supplementary figures [file 41598_2019_41013_MOESM1_ESM.pdf]

Supplementary figures for:

Late Eocene to earliest Oligocene temperature perturbations in the North Sea Basin: interplay of climate and gateways

Kasia K. Śliwińska, Erik Thomsen, Stefan Schouten, Petra L. Schoon, Claus Heilmann-Clausen

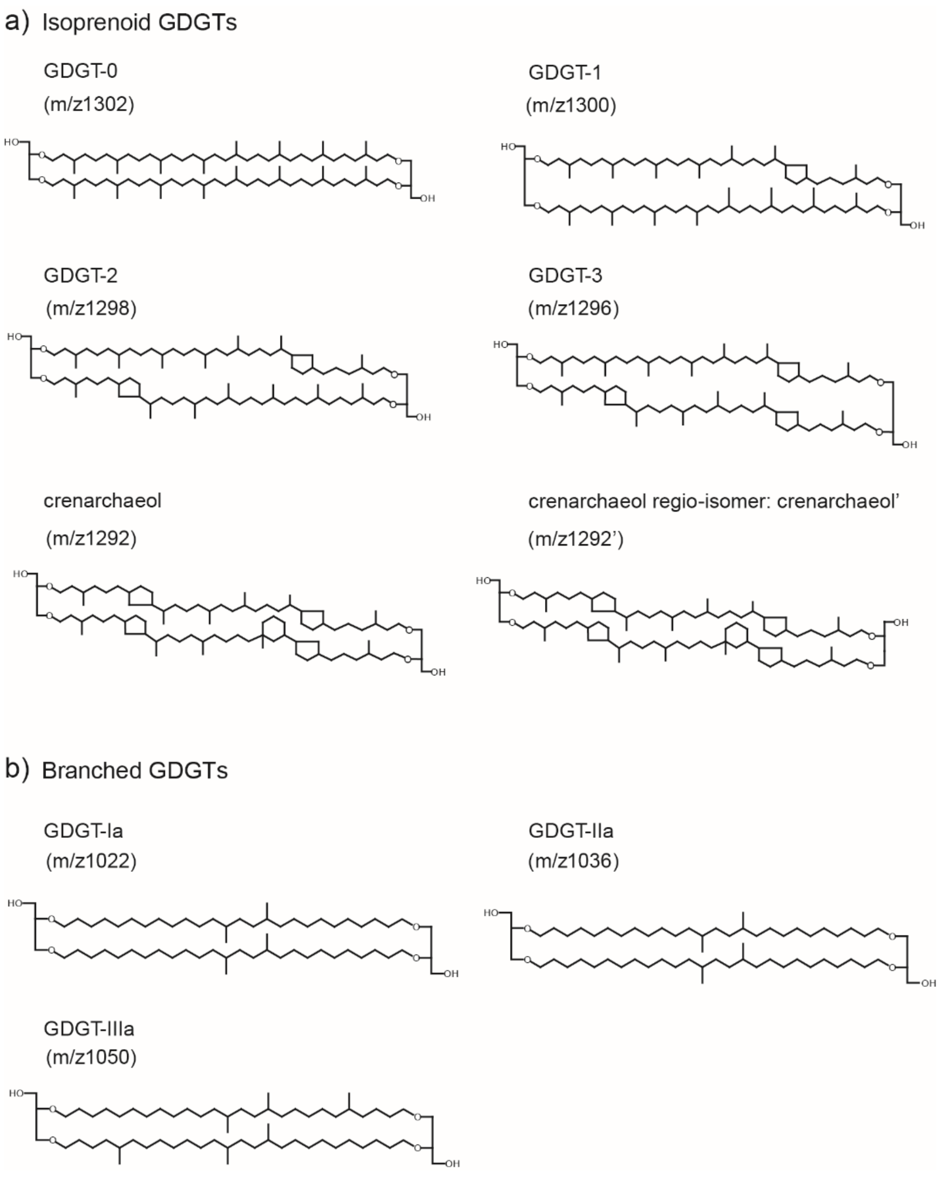

Figure S1 Structures of (a) isoprenoid and (branched) glycerol dialkyl glycerol tetraethers. Associated values of $M+H^+$ ions are given for each lipid.

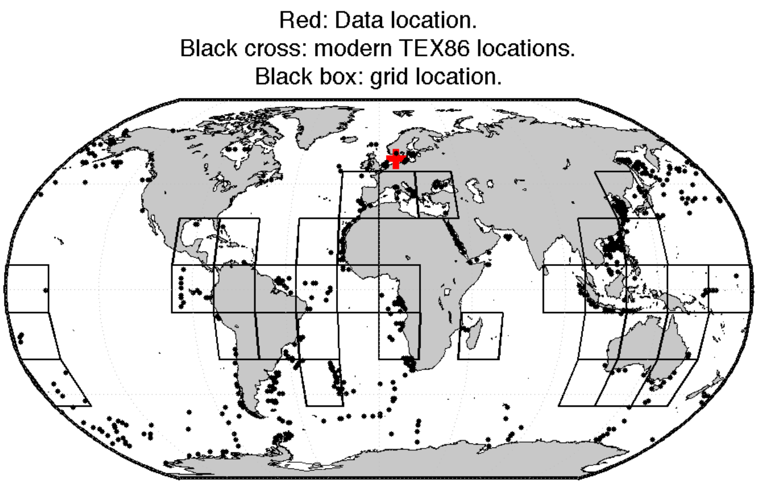

Figure S2 A world map showing the location of the core site, the coretop TEX_{86} data and the modern analog locations as calculated from BAYSPAR

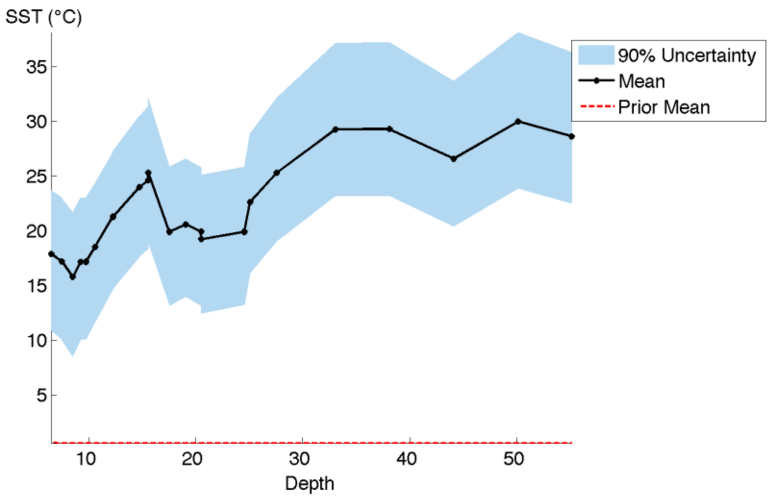

Figure S3 BAYSPAR sea surface paleotemperature predictions. Mean values are in black and the 90% uncertainty intervals is marked in blue. The prior mean is plotted as a dotted red line.

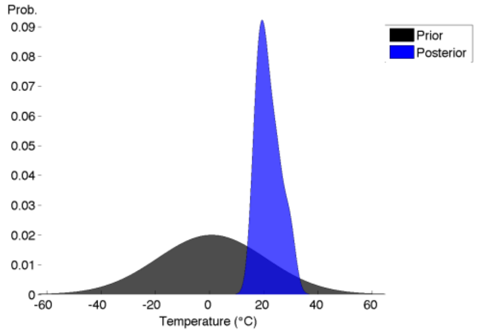

Figure S4 A plot of the prior and posterior temperature distributions, with the mean values removed for the BAYSPAR calibration

**Site**	Kysing-4						Logarithmic		BAYSPAR				Figure 2: main data			
**Depth [m]**	**BIT**	**%GDGT-0**	**MI**	**RI**	**ΔRI**	**fCren':Cren' + Cren**	**TEX^{H}_{86}**	**SST_{TEX86H}**	**TEX_{86}**	**5th**	**50th**	**95th**	**Depth [m]**	**BIT_avg**	**SST_{TEX86H}_avg**	**$SST_{TEX86BAYSPAR}$_avg**
6.50	0.40	48	0.19	2.05	0.09	0.03	0.54	20.3	0.54	10.9	17.9	23.7	6.50	0.4	**20.2**	**17.9**
6.50	0.39	46	0.18	2.11	0.02	0.03	0.54	20.2	0.54	10.9	17.9	23.7				
7.5	0.27	47	0.19	2.10	0.01	0.03	0.53	19.7	0.53	10.1	17.2	23.1	7.50	0.3	**19.6**	**17.2**
7.5	0.27	47	0.20	2.09	0.02	0.03	0.53	19.6	0.53	10.1	17.2	23.0				
8.54	0.23	44	0.17	2.18	-0.13	0.03	0.51	18.4	0.51	8.5	15.8	21.7	8.54	0.2	**18.4**	**15.8**
9.26	0.17	44	0.20	2.17	-0.07	0.03	0.53	19.6	0.53	10.1	17.2	23.0	9.26	0.2	**19.6**	**17.2**
9.26	0.17	46	0.20	2.12	-0.02	0.03	0.53	19.6	0.53	10.1	17.2	23.0				
9.76	0.19	45	0.19	2.14	-0.02	0.03	0.53	19.8	0.53	10.1	17.2	23.0	9.76	0.2	**19.9**	**17.2**
9.76	0.19	46	0.20	2.13	-0.01	0.03	0.53	19.9	0.53	10.1	17.2	23.0				
10.62	0.10	39	0.19	2.36	-0.17	0.04	0.55	21.1	0.55	11.7	18.5	24.4	10.62	0.1	**21.1**	**18.5**
12.27	0.10	40	0.20	2.34	-0.05	0.05	0.59	22.8	0.59	14.7	21.3	27.2	12.27	0.1	**22.9**	**21.3**
12.27	0.10	40	0.20	2.33	-0.04	0.05	0.59	22.9	0.59	14.7	21.3	27.3				
14.75	0.11	36	0.21	2.47	-0.06	0.07	0.63	24.7	0.63	17.6	24.0	30.5	14.75	0.1	**24.8**	**24.0**
14.75	0.10	36	0.21	2.46	-0.04	0.07	0.63	24.8	0.63	17.7	24.0	30.5				
14.75	0.11	37	0.21	2.45	-0.03	0.07	0.63	25.0	0.63	17.7	24.0	30.5				
15.58	0.11	36	0.21	2.48	-0.01	0.07	0.64	25.5	0.64	18.4	24.6	31.4	15.58	0.1	**25.6**	**25.0**
15.58	0.11	34	0.20	2.55	-0.07	0.07	0.65	25.6	0.65	19.0	25.3	32.1				
17.53	0.37	44	0.22	2.21	0.04	0.04	0.57	22.1	0.57	13.2	19.9	25.8	17.53	0.4	**22.1**	**19.9**
17.53	0.36	44	0.23	2.20	0.04	0.04	0.57	22.0	0.57	13.1	19.9	25.8				
19.08	0.38	42	0.23	2.27	-0.01	0.04	0.58	22.5	0.58	14.0	20.6	26.6	19.08	0.4	**22.5**	**20.6**
20.53	0.40	46	0.23	2.14	0.08	0.04	0.57	21.7	0.57	13.2	19.9	25.8	20.53	0.4	**21.7**	**19.6**
20.53	0.41	46	0.24	2.12	0.10	0.04	0.56	21.6	0.56	12.5	19.2	25.1				
24.53	0.28	44	0.22	2.21	0.03	0.04	0.57	22.1	0.57	13.2	19.9	25.8	24.53	0.3	**22.0**	**19.9**
24.53	0.29	44	0.22	2.19	0.04	0.04	0.57	21.9	0.57	13.2	19.9	25.9				
24.53	0.29	44	0.22	2.20	0.04	0.04	0.57	22.1	0.57	13.2	19.9	25.8				
25.08	0.14	37	0.21	2.43	-0.07	0.06	0.61	24.1	0.61	16.2	22.6	29.0	25.08	0.1	**24.1**	**22.6**
25.08	0.14	39	0.22	2.37	-0.01	0.06	0.61	24.0	0.61	16.2	22.6	28.9				
27.58	0.08	36	0.25	2.48	0.03	0.09	0.65	26.0	0.65	19.1	25.3	32.2	27.58	0.1	**26.0**	**25.3**
33.06	0.08	30	0.23	2.69	0.04	0.09	0.71	28.5	0.71	23.2	29.3	37.2	33.06	0.1	**28.5**	**29.3**
38.08	0.08	29	0.23	2.71	-0.01	0.09	0.71	28.3	0.71	23.2	29.3	37.2	38.08	0.1	**28.3**	**29.3**
44.08	0.11	33	0.22	2.58	-0.02	0.07	0.67	26.7	0.67	20.4	26.6	33.7	44.08	0.1	**26.7**	**26.6**
50.08	0.10	30	0.24	2.66	0.10	0.09	0.72	28.8	0.72	23.9	30.0	38.2	50.08	0.1	**28.8**	**30.0**
55.08	0.15	32	0.24	2.59	0.09	0.09	0.70	28.1	0.7	22.5	28.6	36.4	55.08	0.2	**28.1**	**28.6**
**MEAN**	**0.21**	**40**	**0.21**	**2.32**	**0.00**	**0.05**	**0.59**									
						**STDEV**	**0.06**									

Table S1 Data compilation from the Kysing-4 site: TEX86H, branched isoprenoid tetraether (BIT) index, %GDGT-0 index,Methane Index (MI), Ring Index (RI), ΔRI values (see text for details) and the relative abundance of crenarchaeol isomer (fCren':Cren' + Cren). Logarithmic: TEX^{H}_{86} and TEX^{H}_{86} -derived sea surface temperatures (SST_{TEX86H}), BAYSPAR: shows TEX_{86} and the mean paleotemperatures (50th) with 90% of uncertainty. With bold are highlighted figure data with values averaged for duplicated and triplicated sample analysis.
